# Morphological and molecular characterization of *Iotonchus lotilabiatus* n. sp. (Nematoda: Iotonchidae) from Lao Cai Province, Vietnam

**DOI:** 10.21307/jofnem-2021-066

**Published:** 2021-07-27

**Authors:** Tam T. T. Vu, Thi Mai Linh Le, Thi Duyen Nguyen

**Affiliations:** 1 Institute of Ecology and Biological Resources VAST, 18 Hoang Quoc Viet, Cau Giay, Hanoi, Vietnam; 2 Graduate University of Science and Technology VAST, 18 Hoang Quoc Viet, Cau Giay, Hanoi, Vietnam

**Keywords:** Morphology, Ribosomal DNA, Taxonomy

## Abstract

The new species *Iotonchus lotilabiatus* n. sp. from Bat Xat Nature reserve in Lao Cai Province, Vietnam is described and the molecular data (18S and 28S rDNA) are given. Females of the new species are characterized by large body size (*L* = 3.8-5.2 mm); barrel shaped buccal cavity of large size (41–54×70–89 µm) with dorsal tooth apex located 22 to 23.5% of buccal cavity length from its base; reproductive system didelphic-amphidelphic, vagina long, corresponding almost half of body width at the vulva with distinct *parrefringens vaginae*, sclerotized and triangulated pieces in optical section; tail long filiform, ventrally arcuate, with three small caudal glands in tandem and prominent terminal spinneret. Males with spicules 119 to 148 µm in length, and slender, slightly curved gubernaculum, 39 to 44 µm long and with 13 to 14 ventromedian supplements. The new species *Iotonchus lotilabiatus* n. sp. is closest to *I. miamaensis* (Khan and Araki, 2002), but differs by having larger size of body length and buccal cavity, lower position of dorsal tooth apex; longer female tail with lower *c* but higher *c′* ratios and the presence of advulval cuticular structures.

The genus *Iotonchus* was proposed by Cobb (1916) when he split the genus *Mononchus* (Bastian, 1865) into five subgenera *Mononchus*, *Prionchulus*, *Mylonchulus*, *Iotonchus*, *Anatonchus*, and ultimate raised in rank to genus level. The known species became the type species of the genus as *I. gymnolaimus* (Cobb, 1893, 1916). In 1982, Jairajpuri and Khan proposed the new genus *Jensenonchus* based on the type species *J. ovatus* (Jensen and Mulvey, 1968) which was transferred from the genus *Iotonchus*. The genus *Jensenonchus* differs from the genus *Iotonchus* in having more anteriorly dorsal tooth position and a weak longitudinal ridge opposite to dorsal tooth, anteriorly gradually merging into the ventral wall (Jairajpuri and Khan, 1982). Siddiqi (1984a) established the new genus *Mulveyellus* based on the type species *M. jairi* (Lordello, 1959) and five species which were moved from the genus *Iotonchus* including *I. antendontus* (Mulvey, 1963a)*, I. antedontoides* (Coetzee, 1967)*, I. longicaudatus* (Baqri et al., 1978), *I. monhystera* (Cobb, 1916) and *I. vorax* (Mulvey, 1963a). This genus is distinguished from the genus *Iotonchus* by having more anteriorly dorsal tooth position (Siddiqi, 1984a). Recently, Siddiqi (2015) proposed the new genus *Megaiotonchus* (Siddiqi, 2015) from the genus *Iotonchus* based on more anteriorly dorsal tooth position and the presence of dorsal and ventral body pores in the rectal region. The species *Iotonchus ophiocercus* (Andrássy, 1993b; Clark, 1961) became the type species of the genus *Megaiotonchus* and eight species which were transferred from the genus *Iotonchus* including *I. caesar* (Alekseev, 2001), *I. candelabri* (Yeates, 1992), *M. kheri* (Mohadas and Prabhoo, 1979), *I. maragnus* (Clark, 1961), *I. montanus* (Yeates, 1992), *I. percivali* (Clark, 1961), *I. spinicaudatus* (Coetzee, 1967) and *I. vulvapapillatus* (Andrássy, 1964) and two new species as *M. loofi* (Siddiqi, 2015), and *M. nacobbi* (Siddiqi, 2015) were described (Siddiqi, 2015).

Currently, 77 valid *Iotonchus* species have been described with the most recent three being *I. goshiensis* (Khan et al., 2008), *I. hinokumaensis* (Khan et al., 2008), and *I. ogiensis* (Khan et al., 2008). The main diagnostic features of the genus are: (i) body length 0.8 to 5.2 µm long; (ii) medium to large size of buccal cavity, roomy shape, dorsal tooth located at or nearly at the base of buccal cavity, no tooth or denticle on subventral wall; (iii) pharyngo-intestinal junction tuberculate; (iv) female genital system amphidelphic or monodelphic; (v) spicule (if male present) more or less arcuate, bifurcate lateral guiding pieces present; and (vi) tail similar in both sexes, variable in shape and length, predominantly conoid or filiform, caudal glands and spinneret present or absent. At species level, taxa are differentiated using mainly morphometric features such as: tail length or *c′* index, size of buccal cavity, position of dorsal tooth, body length, type of reproductive systems, the degree of development of spinneret, and the position of caudal glands opening.

To date, among predaceous nematodes of the order Mononchida in Vietnam, 17 *Iotonchus* species have been recorded from several provinces (Vu, 2015). In the present study, *Iotonchus lotilabiatus* n. sp. is described based on morphological, morphometric, and molecular data from the Bat Xat Natural Reserve in Lao Cai Province, Vietnam. An updated key to species based on female characteristics and a compendium of all the known species are also provided.

## Material and methods

### Nematode extraction, preservation, and morphological studies

Soil samples were collected randomly from a pristine forest inthe Bat Xat Nature Reserve, Lao Cai Province, Vietnam. Nematodes were extracted from soil samples using modified Baermann funnel technique (Southey, 1986).

They were heat killed, fixed in 4% formaldehyde (for morphological observations) or in a DESS mixture (Yoder et al., 2006) (for molecular analyses), transferred to anhydrous glycerol (Seinhorst, 1962), and mounted on glass slides for microscopic observation. Measurements were performed with a Nikon digital camera on a Nikon Eclipse *Ni* microscope at the Institute of Ecology and Biological Resources, Vietnam Academy of Science and Technology (VAST), Vietnam. Observations of morphological diagnostic features and light microscope were taken with a Nikon digital camera mounted on a Nikon Eclipse *Ni* microscope. Illustrations were drawn using a Nikon Eclipse *Ni* microscope equipped with a Nikon Y-IDT drawing tube. Photographs and illustrations were edited by using Adobe Photoshop CC 2018.

### DNA extraction, polymerase chain reaction (PCR), and sequencing

Nematode DNA was extracted from a single individual as described by Holterman et al. (2006) and DNA extracts were stored at –20° until used as PCR template. The D2-D3 expansion segment 28S rDNA and 18S were amplified using the forward D2A (5′–ACAAGTACCGTGGGGAAAGTTG–3′) and reverse D3B (5′–TCGG AAGGAACCAGCTACTA–3′) primers (Subbotin et al., 2006) and primers 18S (18F : 5′-TCTAGAGCTAATAC ATGCAC-3′/18R: 5′-TACGGAAACCTTGTTACGAC-3′). All PCR reactions contained 12.5 μl Hot start green PCR Master Mix (2x) (Promega, USA), 1 μl of the forward and reverse primer (10 μM each), the 3 μl DNA template and sterile Milli-Q water to 25 μl of the total volume. All PCR reactions were performed in SimpliAmp Thermal cycler (Thermo Fisher Scientific) as follows: an initial denaturation step at 95°C for 4′min, followed by 40 cycles at 95°C for 30 sec, 54°C for 30 sec, and 72°C for 60 sec with a final incubation for 5 min at 72°C. Amplicons were visualised under UV illumination after Simplisafe gel staining and gel electrophoresis. Purified PCR products were sent to Apical Scientific Company for sequencing (Selangor, Malaysia). After sequencing the obtained *Iontonchus lotilabiatus* n. sp. rDNA sequence fragments were deposited in GenBank under the following accession numbers: MW218936-MW218937 (18S rDNA) and MW218934-MW228378 (28S rDNA).

### Phylogenetic analyses

For phylogenetic relationships, analyses were based on 18S and 28S rDNA. The newly obtained rDNA sequences were analysed using the BioEdit sequences avalaible in GenBank using the ClustelW aligment tool implemented in the MEGA 7 version 7.0 (Kumar et al., 2016). The final 18S and 28S rDNA datasets for phylogenetic study included sequences from the current study *Iotonchus lotilabiatus* sp. nov. and available sequences of Mononchida representatives from GenBank. The prepared multiple alignments of 18S and 28S rDNA generated by the ClustalW algorithm were routinely manually edited in order to eliminate improper phylogenetic signals. The phylogenies were constructed with the program MEGA 7 version 7.0. Maximum likelihood with T92+G substitution model for both, 18S and 28S data sets was used.

## Description and discussion

### *Iotonchus lotilabiatus* n. sp.

**Figure 1:**
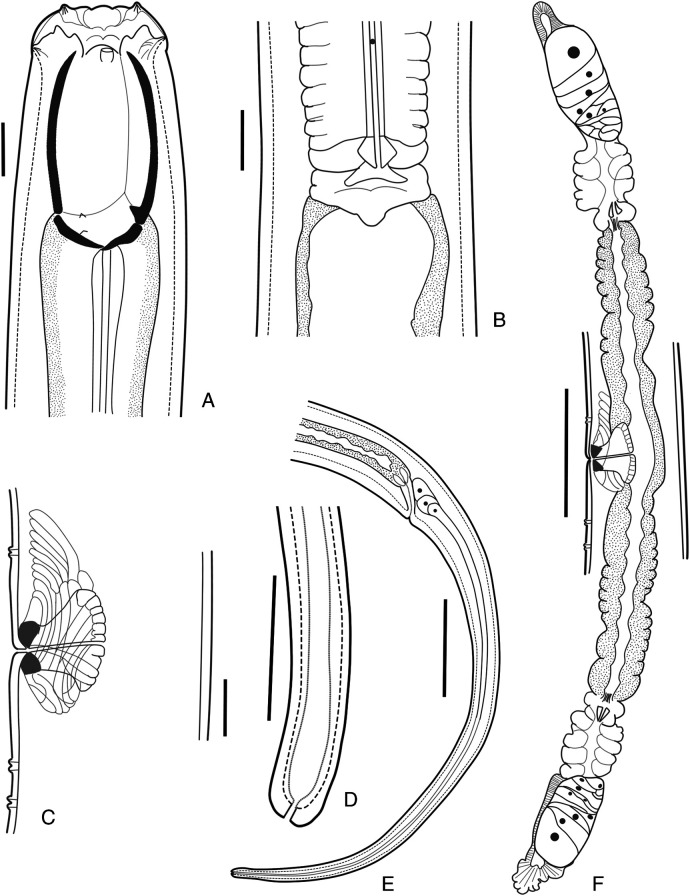
Holotype female *Iotonchus lotilabiatus* n. sp. Female. A. Head region. B. Pharyngo-intestinal junction. C. Vulval region with advulval ventromedian papillary structures. D. Tail terminus. E. Tail region. F. Female reproductive systems. Scale bar: A, B, C, D = 20 µm, E = 100 µm.

**Figure 2:**
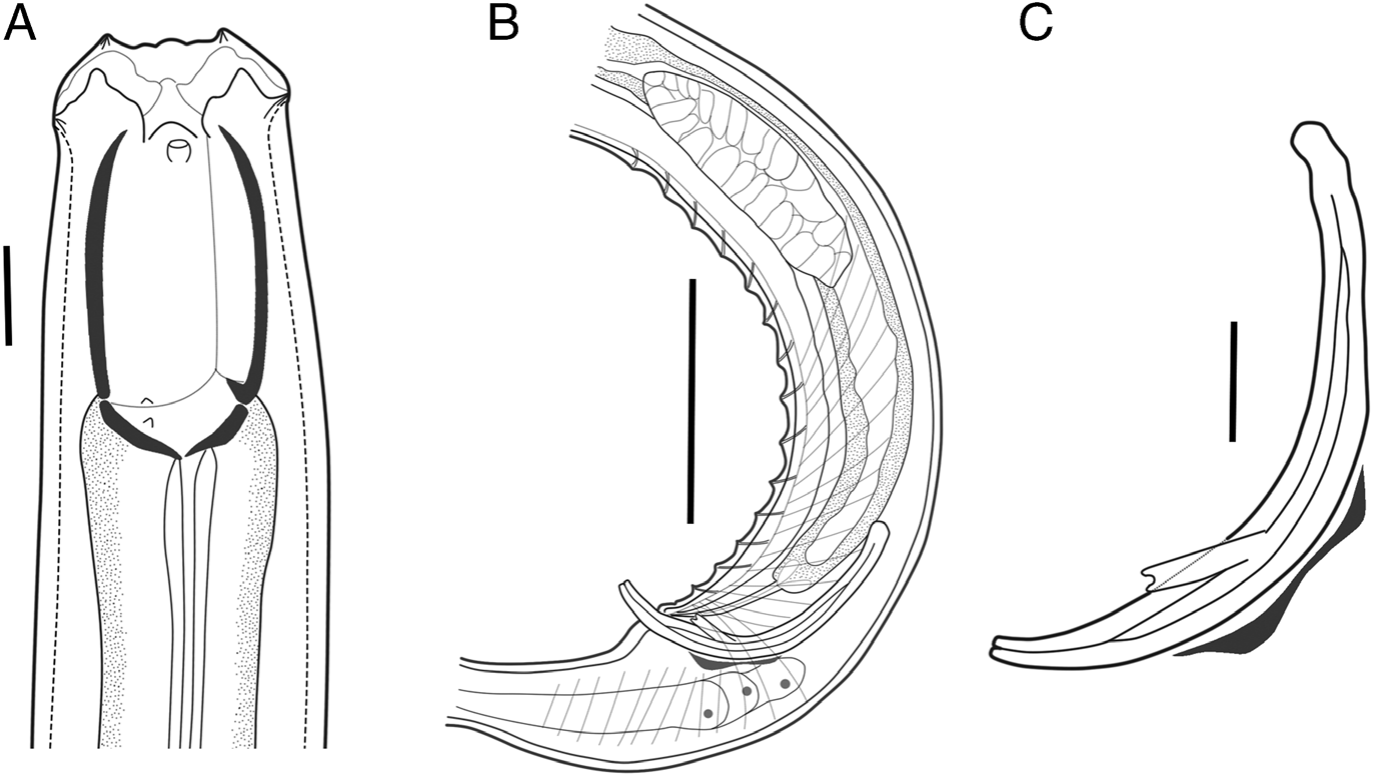
Paratype male *Iotonchus lotilabiatus* n. sp. Male. A. Head region. B. Tail region. C. Spicule, gubernaculum and accessory piece. Scale bar: A, C = 20 µm, B = 100 µm.

**Figure 3:**
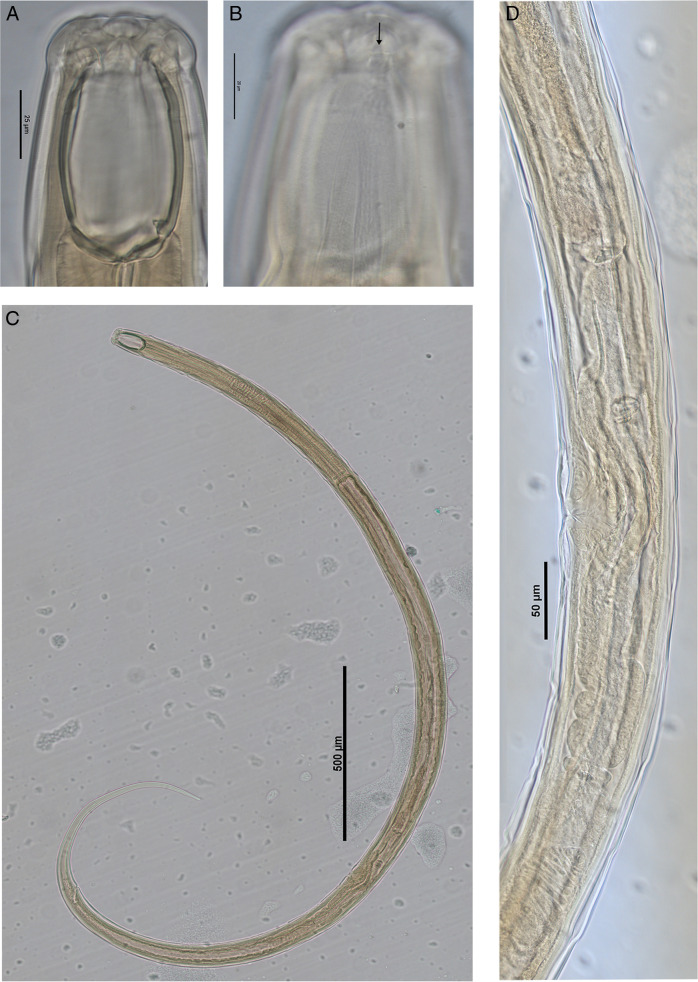
Holotype female *Iotonchus lotilabiatus* n. sp. A. Head region. B. Amphidial aperture. C. Entire body. D. Reproductive system. Scale bars indictaed.

**Figure 4:**
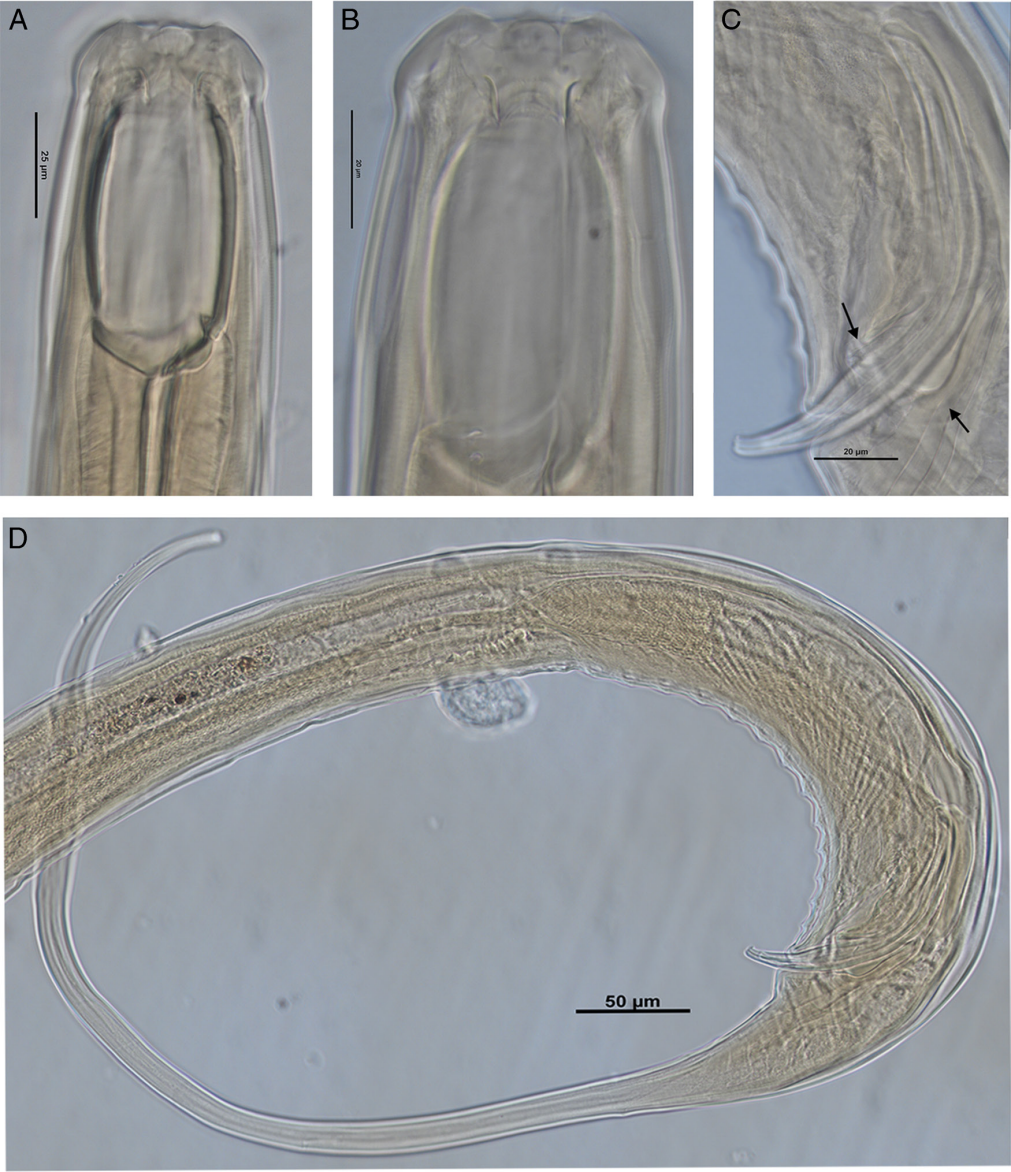
Paratype male *Iotonchus lotilabiatus* n. sp. Male. A. Head region. B. Head region with protruding papillae. C. Reproductive system with spicule, gubernaculum and lateral guiding piece. D. Tail region with ventromedian supplements. Scale bars indicated.

(Figs. 1-4 and Table 1)

**Table 1.** Morphometrics of females and Males of *Iotonchus lotilabiatus* n. sp. from Lao Cai Province, Vietnam.

	Bat Xat Natural Reserve		
Characters/ratios	Holotype	Paratypes	
*n*	1 female	4 females	6 males
*L*	3643	3768–5163 (4519 ± 585)	2950-4230 (3487± 530)
*a*	49.2	49.8-59.2 (56.3 ± 4.4)	44.1-51.5 (48.9 ± 2.8)
b	4.8	4.9-5.4 (5.1 ± 0.2)	4.7-5.4 (5.0 ± 0.3)
c	6.4	4.5-6.3 (5.6 ± 0.8)	5.3-5.9 (5.6 ± 0.2)
c′	12.2	14.4-19.0 (15.7 ± 2.2)	8.2-13.1 (10.4 ± 1.7)
V (%)	58.3	54.2-59.4 (55.9 ± 2.4)	–
Lip region height	16.5	18.0-21.0 (19.8 ± 1.5)	14.5-17.5 (16.3 ± 1.1)
Lip region width	50.0	52.5-70 (59.5 ± 7.5)	46-52 (48.3 ± 2.7)
Buccal cavity length	73	69.5-88.5 (79.6 ± 8.9	61.5-67.2 (64.1 ± 2.2)
Buccal cavity width	41.5	41.0-54.0 (46.9 ± 6.1)	36.0-41 (37.9 ± 1.6)
Position of tooth apex from the base of buccal cavity	16.7	16.5-19.5 (17.7± 1.5)	14-17 (15.5 ± 1.5)
Nerve ring from anterior end	190	210-244 (226.4± 15.1)	154-204 (174.9 ± 20.0)
Excretory pore from anterior end	225	224-271 (253 ± 22.2)	180.8-232 (204 ± 21.8)
Pharynx length	761.0	770-975.7 (870 ± 98.5)	627.7-789.3 (693.2 ± 70.9)
Anterior branch of genital system	341	341-477 (424 ± 37)	–
Posterior branch of genital system	314	314-462 (406 ± 38)	–
Maximum body width	74.0	75.5-87.0 (80.0 ± 5.1)	60.0-82.0 (71.0 ± 8.0)
Anal body width	46.8	48.0-58.0 (52.0 ± 4.4)	55.0-63.0 (59.8 ± 3.3)
Rectum/cloaca length	49.2	49.0-53.0 (51.5 ± 1.8)	55-64 (59.5 ± 3.4)
Vagina length	34.0	36.5-38.5 (37.7 ± 1.0)	–
Spicule length	–	–	130-141 (136.8 ± 4.1)
Spicule width at widest part	–	–	8.0-11 (9.3 ± 1.4)
Lateral guiding piece length	–	–	17.5-23.5 (19.6 ± 2.9)
Gubernaculum length	–	–	39.5-44 (42.3± 1.7)
Number of supplements	–	–	13-14
Distance from cloacal opening to posteriormost supplement	–	–	20.5-23 (21.4 ± 1.0)
Distance from cloacal opening to anterior most supplement	–	–	201-241.5 (218.9 ± 15.8)
Tail length	572	694-974 (818 ± 125.3)	500-798 (623 ± 114)

**Notes:** All measurements are in µm except where indicated. *a*  =  body length/maximum body width; *b* = body length/pharyngeal length; *c* = body length/tail length; *c′* = tail length/body width at anus; *V* =  (distance from anterior end to vulva/body length)x100. *L* = body length.

### Type material

Holotype female, four paratype females and six paratype males in good condition of preservation. Holotype female on slide *Iotonchus lotilabiatus* n. sp. No. 1; four paratype femaleson slides *Iotonchus lotilabiatus* n. sp. No. 2-5, and six paratype males on slides *Iotonchus lotilabiatus* n. sp. No. 6-11 in permanent mounts in glycerin. All slides have been deposited in the nematode collection at the Department of Nematology, Institute of Ecology and Biological Resources, Vietnam Academy of Science and Technology (VAST), Vietnam.

### Type habitat and locality

Soil around the roots of random forest trees in the Bat Xat Nature reserve (N = 22°37′14″ and E = 103°37′24″, altitude 1,900 m), Bat Xat District, Lao Cai Province, Vietnam.

### Etymology

The specific epithet refers to the morphology of the labial region (lips lotus-petals shaped) that characterizes this species.

Measurements: see Table 1.

### Description

#### Females

Large size of nematodes. Body ventrally arcuate after fixation. Body tapering slightly anterior to vulva position but more sharply toward posterior end. Maximum body width at the level of vulva. Cuticle smooth, 4.7 (3.4-5) µm thick at the base of pharynx, sub-cuticle distinctly striated. Labial region offset by a depression from the body contour, 2.8 to 3 times as wide as high. Labial region slightly separated six lips in the shape of lotus-petals with prominent labial papillae. Anterior sensilla arranged in two circles: an anterior one of six inner labial papillae, posterior crown with six outer labial papillae and four cephalic papillae protruding beyond the body outline. Buccal cavity begins at 19-22 μm from the anterior end of the body, large size, 1.6-1.8 as long as wide, barrel shaped, its wall strongly sclerotized, vertical plates parallel, basal oblique plates flattened. Amphidial fovea small, cup-shaped, with oval aperture 4-5 μm wide, located around at the beginning of buccal cavity or slightly lower. Dorsal tooth small size, located at the base of vertical plates, apex pointed forward, at 22.5 (21.6-23.4) % its length from the base of buccal cavity. Two small but prominent foramina present at the base of the buccal cavity. About the posterior fifth of buccal cavity embedded in pharyngeal tissues.

Body diameter length at the pharynx base about 1.2 to 1.4 times head width. Pharynx cylindrical, surrounded by the nerve ring, located at 26 (24-27) % of its length, measured from the anterior body end, respectively. Secretory-excretory (SE) pore conspicuous, situated just posterior to nerve ring at 29 (28-30) % of its length from anterior body end. Pharyngo-intestinal junction tuberculate; cardia with conoid projection into wide intestinal lumen. Distance between pharynx base and vulva 1.7 to 2.0 times of the pharynx length. Rectum slightly curved, as long as anal body width. Tail long filiform, 0.7 to 1 mm long, ventrally curved, correspondence 16 to 22% of body length or 12.2 to 19.0 anal body diameters long; three small caudal glands in tandem and opening on terminal tail.

Genital system typical for the genus, didelphic-amphidelphic, both branches about equally developed: anterior branch occupies 9.4 (8.9-10.4) % of body length and posterior branch occupies 9.0 (8.5-10.2) % of body length. Ovaries reflexed, 100 to 145μm long in anterior branch and 85 to 120 μm long in posterior branch; oocytes arranged in a row except at its tips. The uterus is relatively long, 170 to 240 μm long in each branch; sphincter present at the oviduct-uterus junction, valve sclerotized. Oviduct length 133 to 203 μm in anterior branch and 123 to 200 μm in posterior branch, with well-marked *pars dilatata*, connected to the uterus with a muscular sphincter. Vulva a transverse slit, situated on the posterior half of the body. Zero to one pre- and one to two conspicuous post advulval papillae. Vulva-anus distance 1.4 to 1.7 times its tail length. Uterus in one paratype female with two large eggs (177 μm long × 63.4 μm wide in anterior branch and 200.4 μm long × 61 μm wide in posterior branch). Vagina length corresponds to almost half of body width. *Pars proximalisvaginae* as long as wide, gradually expanded in distal part, surrounded by strong circular muscles. *Pars refringensvaginae* appearing as two prominent, triangular sclerotized pieces (6.7×7 µm) in longitudinal optical section. *Pars distalisvaginae* short. Vulva transverse in ventral view.

#### Males

Males are similar to females in general morphology, but more strongly curved to coiled in the posterior region. Buccal cavity smaller than females, 36–41×61.5–67 μm. Genital system diorchic, testes pared, outstretched and opposed, 1.2 to 1.7 mm long. Spicules slender, ventrally arcuated with bifurcate terminus, 2.1 to 2.4 times longer than body diameter at cloacal aperture and 8 to 11 μm long at the widest part. The head of spicule round-shape, 13 to 19 μm long and 7.5 to 9.5 μm wide; offset by shallow depression. The median piece is 104 to 115 μm long and 3.5 to 4.5 μm wide; round blade part. Lateral guiding pieces straight with bifurcate terminus, 18 to 24 μm long. This furcation is symmetrical and well-marked. Gubernaculum is well developed, 39 to 44 µm long (following Peña-Santiago et al., 2014). Ventromedian supplements 13 to 14 in number, conical and regularly arranged, occupies 5 to 7.7% of the body length. The most anterior supplement is situated at 201 to 242 µm from cloacal aperture. The distance between the first and last supplement is 178 to 220 µm. Male tail is 500 to 800 µm long, slightly shorter than the female tail.

### Diagnosis

*Iotonchus lotilabiatus* n. sp. is characterized by large adult body size (3.8-5.2 mm); long length of buccal cavity (69.5-88.5 µm); posterior position of dorsal tooth apex (21.6-23.4)% of buccal cavity length from its base; didelphic-amphidelphic with both branches equally developed, *pars refringens vaginae* sclerotized (6.7×7 µm), intriangulated shape; the presence of small ventromedian papillary structures on the vulval region; the presence of males with long spicules (119-148 µm) with rounded head and blade part, gubernaculum (39-44 µm) and lateral guiding piece (17-24 µm) well developed; 13 to 14 ventromedian supplements.

In general appearance *Iotonchus lotilabiatus* n. sp. is similar to *I. apapillatus* (Dhanam and jairajpuri, 1998), *I. brachylaimus* (Cobb, 1917), and *I. miamaensis* (Khan and Araki, 2002) based on the morphological characters as: a large body length (*L*>3 mm), long length of buccal cavity (>65 µm), didelphic-amphidelphic, caudal glands opening on terminal tail and the presence of males. From *I. apapillatus*, it differs by having larger body length (3.8-5.2 vs 2.7-3.5 mm), slenderer nematodes (*a* = 49-59 vs 35-39); larger buccal cavity (41-54×70–89 μm vs 40-44×68-70 μm); lower *c* value (4.5-6.3 vs 10-13) but higher *c′* value (12.2-19 vs 4.3-5.8); vulval more anterior (*V* = 54.2-59.4% vs *V* = 62-74%), the presence of advulval cuticular structures and the number of ventromedian supplements in males (13-14 vs 17-19). The new species *Iotonchus lotilabiatus* n. sp. differs from *I. brachylaimus* by having larger body length (3.8-5.2 vs 3.2 mm), slenderer nematodes (*a* = 49-59 vs 33); longer length of buccal cavity (70-89 μm vs 64 μm); lower *c* value (4.5-6.3 vs 17) but higher *c′* value (12.2-19 vs 3.7); the presence of advulval cuticular structures; longer spicule length (130-141 vs 95 μm) and the number of ventromedian supplements (13-14 vs 16).

From *I. miamaensis*, it is distinguished by having larger size of body length (3.8-5.2 vs 3.2-3.5 mm); larger size of buccal cavity (41-54×70-89 μm vs 42-45×64-72 μm), more posterior dorsal tooth apex (21.6-23.4% vs 24-27%); longer female tail (0.57-0.97 vs 0.4-0.43 mm), lower *c* (4.5-6.3 vs 7.6-8.4) but higher *c′* (12.2-19.0 vs 7.5-8.3) ratios and the presence of advulval cuticular structures.

Table 2 presents a compendium of *Iotonchus* species morphometrics for comparative purposes (However, five species were not included in Table 2 and the key because of unavailable literatures*: I. brachylaimium* (Aboul-Eid and Ameen, 1982); *I. kvavadezi* (Eliava)*; I. longisaccatus* (Mohilal et al., 2000); *I pharoensis* (Aboul-Eid and Ameen, 1982), and *I. terminus* (Mohilal et al., 2000).

**Table 2.** Compendium of the species belonging to the genus *Iotonchus* (Cobb, 1917).

Species	*n*	*L*	*a*	*b*	*c*	*c'*	*V*	B.C.L.	B.C.W.	%DT	Spinneret	Female gonad	Spicule length	Number Suppls	Country	Reference
*abrahmami*	9♀♀	2.7-3	40-51	4.6-5.0	5.8-12.3	5.9-12.8	64-72	42-47	27-31	33-36	Terminal	Pseudo-prodelphic			Malaysia	Loof (2006)
	2♂♂	2.7-2.8	54	4.7-4.8	6.8	8.3-8.5		42-43	25-26				90-91	11		
*acuticaudus*	11♀♀	1.9-2.1	29-34	3.8-4.7	9.1-11.8	6.1a	71-75	40-50	28-32	23a	Subdorsal	Monodelphic			Nigeria	Mulvey and Jensen (1967)
	11♂♂	2.0-2.4	27-34	4.0-4.4	9.5-11.8	4.3a		40-45	27-31				78-88	6-8		
*Acutus*	10♀♀	1.8-2.2	32-39	5.3-6	12-15.5	4.3a	61-66	37-38	24-26	28a	No	Didelphic			South Africa	Heyns and Lagerwey (1965)
	10♂♂	1.4-1.9	28-40	4.4-5.5	13.2-15.6	2.7a		26-37	17-23				67-83	9-12		
	4♀♀	1.7-2.4	31-41		13.6-18	3.9a	63-66	39-43	20-27		No	Didelphic			USA	Mulvey (1963a)
*aequabilis*	?♀♀	2.2-2.5	36-43	5.4-5.6	34-41	1.3-1.8	70-72	44-48	28-33		No	Didelphic			Ukraine	Susulovsky (1998)
*aequatorialis*	5♀♀	2.2-2.4	20-30	4.4-4.7	4.3-4.8	8.3-10	51-54.4	57-65	32-38.5	23a	Terminal	Didelphic			Ecuado	Vinciguerra and Orselli (2006)
*anisostomus*	1♀	1.72	30	3.7	12.9	5a	68	40	17	23a	Terminal	Monodelphic			Thailand	Buangsuwon and Jensen (1966)
*arcuatus*	8♀♀	1.7-1.8	35-41	4.4-4.7	4.9-6	9.2-10.5	55-57	38-41	24-28	24-34	No	Didelphic			Japan	Khan et al. (2000)
*apapillatus*	8♀♀	2.7-3.5	35-39	4.4-4.7	10-13	4.3-5.8	62-74	67-80	40-44	19-27	Terminal	Didelphic			India	Dhanam and Jairajpuri (1998)
	3♂♂	2.9	41	4.6-4.7	11-15	2.9-4.3		64-67	34-35				125-138	17-19		
*baqrii*	9♀♀	1.5-1.7	28-37	4.0-4.6	5-7	12.2a	62-70	32-37	28-32	29-34	Subventral	Monodelphic			India	Jairajpuri (1969)
	9♂♂	1.5-1.7	31-35	4.3-4.8	5-7	6.8a		28-32	24-26	25-28			80-93	9-10		
*basidontus*	6♀♀	1.7-2.4	25-32	3.7-4.3	6.5-8.6	6.6a	55-60	48a	30a	25	Terminal	Didelphic			New zealand	Clark (1960)
	1♂	2	30	3.9	8.6	4.8a							83a	14		
*brachylaimus*	1♀	3.2	33.3	4.8	17	3.7a	65	64a	53a		Terminal	Didelphic			USA	Mulvey (1963a), (after Cobb, 1917)
	1♂	3.5	40	5.3	17								95	16		
*carpathicus*	13♀♀	2.5-3.5	31-44	4.2-5.1	4.3-6.6	12.1a	56.5-60	46-62	36-52	23-35	No	Didelphic			Romania	Popovici (1990)
	16♂♂	2.1-3.7	33-47	4.3-5.1	4.9-6.7			47-57	33-50				72-100	11-13		
*chantaburensis*	4♀♀	0.8-1	24-32	3.5-4.5	3.8-4.9		59-64	22-25	9-14		Terminal	Monodelphic			Thailand	Buangsuwon and Jensen (1966)
	6♀♀	0.9-1	24-26	3.8-3.9	5.8-6	6.7-7.4	63-65	26-30	14-17		Subventral	Monodelphic			India	Mohadas and Prabhoo (1979)
*clarki*	6♀♀	1.7-2	28-34	3.9-5.2	4.6-5.9	8.8a	52-58	40-43	23-29	20a	No	Didelphic			Nigeria	Mulvey and Jensen (1967)
*consimilis*	?♀♀	2.8-3.5	34-41	4.5-4.9	6.6-7.6	7.3a	68-69	60-63	37-40		Terminal	Monodelphic			Brazil, Hawaii	Mulvey (1963a), (after Cobb, 1917)
*cucumis*	5♀♀	2.9-3.2	39-44	4.2-4.3	10.7-12.2	4.7-5.2	64-66	57-60	34-39	23	Terminal	Didelphic			Korea	Khan et al. (2002)
*cuticaudatus*	12♀♀	2.8-3	30-37	4.6-5.8	7-10	5.5-7.8	62-64.5	57-65	33-37	22-25	Terminal	Didelphic			India	Jana et al. (2007)
	7♂♂	2.3-3.1	30-39	4.4-5.4	9.6-14.5	3.1-3.6		50-57	26-30				123-133	11-15		
*damsanensis*	3♀♀	1.6-1.7	20-22	2.9-3	17-1.7	1.7-2.2	71-75	58-61	35-37	29-32	No	Didelphic			Korea	Choi and Khan (2000)
*devius*	1♀	2.4	37.5	4.2	6.5	9.5	69	54	30	26	No	Monodelphic			Côte d’Ivoire	Siddiqi (2001)
*geminus*	8♀♀	1.7-2.1	25-30	3.9-4.5	12.4-14	3.4a	65-68	38-43	22-24	29a	Terminal	Didelphic			South Africa	Heyns and Lagerwey (1965)
	7♂♂	1.7-1.9	28-33	4.0-4.8	14.6-17.5	2.5a		37-39	18-20				77-88	11-14		
*globibucca*	?♀♀	2.4	31	5	7.1	8.2	59	54	37		No	Didelphic			India	Dhanam and Jairajpuri (2002)
*goshiensis*	4♀♀	1.5-1.6	28-30	3.7-4.1	24-26	1.7-1.9	63-65	40-42	26-27	24-25	No	Didelphic			Japan	Khan et al. (2008)
*guineae*	1♀	2	33	4	7.1	7.1	70	58	34	20a	Terminal	Monodelphic			Guinae	Siddiqi (2001)
	2♂♂	1.9-2.0	35-43	3.9-4.7	5.9-6.9	6.9-8.3		47	26-27				74-89	4-6		
*gymnolaimus*	1♀	2.9	40	4.2	7.1	9a	67	57	40		Terminal	Monodelphic			Fiji, Brazil, USA	Mulvey (1963a), (after Cobb, 1917)
*hinokumaensis*	4♀♀	2-2.2	33-44	4.7-5.4	9.4-10.8	5.3-6.1	60-63	37-40	24-26	26-27	No	Didelphic			Japan	Khan et al. (2008)
	3♂♂	2.1-2.3	41-44	4.4-5.4	8.7-9.3	5.9-6.3		38-40	24-25				68-75	9-11		
*indicus*	28♀♀	1.5-2	21-32	4.0-4.8	5-8	10a	57-65	40-47	28-32	23-25	Subventral	Didelphic			India	Jairajpuri (1969)
*kilumicus*	4♀♀	2.6-2.7	50-57	4.9-5.2	4.0-4.5	17-18	53-55	43-48	23-25	23-27	Subventral	Didelphic			Cameroon	Siddiqi (2001)
	3♂♂	2.4-2.5	49-57	4.9-5.1	4.1-4.6	13-14.6		41-43	18-19				63-73	10-11		
*kirbyi*	5♀♀	3-3.3	40-46	4.2-4.6	6.8-7.8	7.8-9.1	67-72	58-64	32-33	26a	Terminal	Pseudo–prodelphic			Fiji	Siddiqi (1984)
	4♀♀	2.8-3.3	37-40	3.6-4.2	13.5-17.8	3.1-4.2	72-74	60-62	31-33							
*kirghistanicus*	?♀♀	1.4-1.8	20-23	3.2-3.7	23-28	1.5-2	72-75	42	30		No	Didelphic			Kirghistan	Sultanalieva (1983)
	?♂	1.6	19-22	3.3-3.8	20-25									22		
*koupensis*	4♀♀	2.1-2.5	30-36	4.1-4.2	4.1-5.5	8-11	54-58	57-60	29-30	19-20	Subventral	Didelphic			Cameroon	Siddiqi (2001)
	1♂	1.8	29	4.5	5.8	7.2		49	24				90	12		
*lacuplanarum*	2♀♀	2	39-45	4.6	6.1-6.2	10.2	63-64	36-37	26	24-25	Subventral	Monodelphic			New Caledonia	Yeates (1992)
	3♂	1.8-2.1	38-45	4.2-4.9	6.1-7.2	7.4-8.9		32-35	23-29	27-29			47-57	9-12		
*lamottei*	4♀♀	1.6	32-35	4.1-4.4	7.2-7.9	6.4a	65-66	33-41	25-27	22a	No	Monodelphic			Côte	Malcevschi (1981)
	1♂	1.6	31	4	8.3	4.1a		35	22				50	9	d’Ivoire	
*litoralis*	2♀♀	2.1-2.3	24-26	5	10-11	4.5a	61-62	61a	42a	25	Terminal	Didelphic			South	Coetzee (1967b)
	2♂♂	2.6-2.8	36-37	4-5	16	2.7a							104-112	15-17	Africa	
	1♀	2.7	34	4.1	15.1	3.6	68	54	39		Terminal	Didelphic			South Africa	De Bruin and Heyns (1992)
	2♂♂	2.6-2.8	35-37	4.4-4.7	18.4-21	2.2-2.6		49	32-35				107-111	13-14		
*longisaccus*	2♀♀	1.8-2.4	32-38	4.1	9.1-10.9	5.2-5.4	71	50-60	25-31	28a	Terminal	Monodelphic			Côte	Siddiqi (2001)
	1♂	2.35	47	3.9	7.3	7.8		54	25				88	4	d’Ivoire	
*loteniae*	3♀♀	1.9-2	34-42	4.3-5.1	7.3-8.6	6.8-9.5	61-68	30-38	22-25	29a	No	Didelphic			South Africa	De Bruin and Heyns (1992)
*lotilabiatus*	5♀♀	3.8-5.2	50-59	4.9-5.4	4.5-6.3	12-20	54-59	70-88	41-54	22-23	Terminal	Didelphic			Vietnam	Currently paper
	6♂♂	3-4.2	44-52	4.7-5.4	5.3-5.9	8.2-13		62-67	36-41	21-25			130-141	13-14		
*magyar*	1♀	4.2	38	4.9	86	0.7	69	51	26	40	No	Didelphic			Hungary	Andrássy (1973)
*mboticus*	2♀♀	2.2-2.3	42-44	5.2-5.3	4.2-4.3	14-14.7	52-53	40	23	23a	Subdorsal	Didelphic			Cameroon	Siddiqi (2001)
	3♂♂	2.0-2.1	46-47	5.3-5.6	4.0-4.4	12-13.5		37-39	19-20				56-62	10		
*miamaensis*	9♀♀	3.2-3.5	44-50	4.6-5.0	7.6-8.4	7.5-8.3	60-61	64-72	42-45	24-27	Terminal	Didelphic			Japan	Khan and Araki (2002)
	5♂♂	2.8-3.1	45-48	4.5-4.7	11.2-14	3.5-4.7		57-62	34-40				127-138	12-13		
*microdontus*	4♀♀	1.2-1.3	29-31	3.7-3.9	5.7-6		61-64	34-36	19-20		Terminal	Monodelphic			Singapore	Thong (1970)
*muneris*	3♀♀	1.5-1.6	33-36	4.1-4.4	4.1-4.7	11-13.7	58-62	36-37	19-20	17-19	Subventral	Monodelphic			Cameroon	Siddiqi (2001)
*nayari*	13♀♀	2.3-2.7	31-34	4.2-4.6	12-16	4.0-4.9	62-67	55-64	34-38	27-28	Terminal	Didelphic			India	Mohadas and Prabhoo (1979)
	4♂♂	2.2-2.5	31-33	4.1-4.6	13-16.5	2.5-2.9		52-54	28-30				117-126	15-16		
	8♀♀	2.3-2.7	27-34	4.0-4.6	10-12		65-70	51-54	32-36		Terminal	Didelphic			India	Khan and Jairajpuri (1980)
	10♂♂	2.3-2.6	30-37	4.1-4.7	10-13								112-131	12-16		
*ndu*	8 ♀♀	1.8-2.7	39-47	4.3-4.9	4.3-6.9	8-15	50-57	46-54	21-27	24-26	Subventral	Didelphic			Cameroon	Siddiqi (2001)
	3♂♂	1.8-2.2	42-45	4.3-4.8	4.4-6.9	8.9-11.7		45-50	21-22				66-75	10-12		
*nepotum*	7♀♀	1-1.2	22-25	3.1-3.8	13-16	2.2-2.8	66-70	36-40	22-23	17-20	Terminal	Monodelphic			Papua New Guinea	Andrássy (2008)
*nigeriensis*	11♀♀	1.6-1.9	25-35	3.9-4.6	10-13	5.4a	68-72	36-40	21-29	23a	No	Monodelphic			Nigeria	Mulvey and Jensen (1967)
	11♂♂	1.4-1.7	28-36	3.9-4.5	12-16	3.3a		32-35	20-23				63-70	6-8		
*ogiensis*	4♀♀	2-2.3	33-36	4.1-4.4	18-20	2.7-3.3	62-63	38-41	24-26	24	No	Didelphic			Japan	Khan et al. (2008)
	2♂♂	2.1-2.3	32-34	4.4-4.6	14-15	2.5-2.7		45-47	25-28				90-95	12-14		
*obtusus*	1♀	2.8	33	4.2	61	1	68	61	45	21	No	Didelphic			Korea	Choi et al. (1999)
*onchus*	2♀♀	2.4-2.5	35-36	4.6-4.8	7-8	7	61	55	33-35	26-28	No	Didelphic			Korea	Jairajpuri et al. (2000)
	1♂	2.1	34	4.6	8	5		50	30	23			82	11		
*parabasidontus*	7♀♀	2.2-2.6	26-37	4.3-4.9	7.2-8.7	6.9a	56-60	48-55	29-32	23a	Terminal	Didelphic			Nigeria	Mulvey and Jensen (1967)
	5♂♂	2.0-2.4	29-38	4.3-4.7	9.1-11.2	4.2a		42-50	25-28	26a			90	13-15		
	1♀	1.7	24.4	4.3	6.6	6.5	58	49	26						Cameroon	Siddiqi (2001)
*paracutus*	2♀♀	1.9-2.2	23-26	5-5.1	9.4-10.4	3.5-4.6	62-63	57-68	35-40	23a	No	Didelphic			Italia	Vinciguerra and Orselli (2000)
	2♂♂	1.6	23-24	4.6	10-11.3	2.8-3.0		52.5	30-32.5				57.5-66	10-11		
*parageminus*	31♀♀	1.8-2.4	39-53	5.1-6.2	17-24.9	2.5-4.3	63-69	29-40	20-27	25-37	Subdorsal	Didelphic			Spain	Jiménez-Guirado (1994)
	20♂♂	1.7-2.2	36-53	5.1-6.1	24-32	1.6-2.2		27-34	18.5-21.5				53.5-67	9-13		
*paratrichurus*	5♀♀	1.4-1.5	27-35	4.2-4.6	3.6-4.1	13.5-15	55-58	30-32	15.5-17	23-25	Subventral	Monodelphic			Cameroon	Siddiqi (2001)
	2♀♀	1.3-1.4	23-25	4.3-4.5	3.1-3.9	10.3-13		30	17-19							
*pauli*	5♀♀	1.3-1.6	35-42	4.8-5.1	8.4-9.3	6.2a	59-63	29-30	18-19	30a	No	Didelphic			South Africa	Heyns and Lagerwey (1965)
	6♂♂	1.3-1.4	38-45	4.6-5.1	10-11.2	3.7a		26-29	14-15				52-53	8-9		
*pseudodigonicus*	6♀♀	1.4-1.6	31-34	4.2-4.7	4.1-4.7		60-63	36-39	25-27	31-36	Subventral	Pseudo-prodelphic			India	Ahmad and Jairajpuri (1983)
	4♂♂	1.4-1.7	33-41	4.4-4.7	4.6-5.1								60-64	6-8		
*pusillus*	14♀♀	0.7-1	24-30	3.6-4.3	7.0-8.1	4.7-6.9	63-67	22-24	10-13	20-29	No	Monodelphic			Malaysia	Loof (2006)
*recessus*	1♀	2.1	43	4.6	5.8	11	63	38	26	26	Subventral	Monodelphic			New Caledonia	Yeates (1992)
*rayongensis*	1♀	2	37	4.9	9.5	7.7a	57	46	22	33a	Subdorsal	Didelphic			Thailand	Buangsuwon and Jensen (1966)
*rinae*	5♀♀	1.5-1.8	30-38	5	14-15	4.2a	68-72	34a	22a	29	Terminal	Didelphic			South	Coetzee (1967b)
	12♂♂	1.7-1.9	31-40	5	15-19	3.2a							60-70	11-12	Africa	
	10♀♀	1.4-1.9	27-35	3.9-4.5	10-13.3	3.6-5.1	61-70	29-39	18-24		Terminal	Didelphic			South Africa	De Bruin and Heyns (1992)
	8♂♂	1.3-1.7	28-37	4.1-5.1	14-17.7	2.5-2.8							66-74	6-11		
*risoceiae*	1♀	3.3	33	4.6	8.9	6.2a	64	58	47	29a	Terminal	Didelphic			South Africa	Heyns and Lagerwey (1965)
	4♀♀	2.7-3.4	34-37	4.5-4.7	8-10	6.3a	60-64	53-64	32-40		Terminal	Didelphic			India	Khan and Jairajpuri (1980)
	?♂♂	3.1-3.7	36-39	4.5-5.1	11									14-18	India	Ahmad and Jairajpuri (2010)
*rotundicaudatus*	29♀♀	2.3-2.8	45-63	5.9-7.7	55-108	0.8-1.4	62-69	32-45	23-25	25-32	No	Didelphic			Spain	Peña Santiago and Jiménez–Guirado (1991)
*sacculatus*	3♀♀	1.7-2.1	28-35	4.4-4.5	7.6-8.0	6.3-6.6	70-72	53-55	30-31	13-15a	Subdorsal	Monodelphic			Guinea	Siddiqi (2001)
	3♂♂	1.5-1.8	35-39	3.9-4.5	6.8-8.2	5.3-5.5		40-49	22-26				76-79	8-9		
*sagaensis*	4♀♀	2.5-2.9	40-42	4.1-4.6	9.3-11	5.3-6	60-65	58-60	38-40	23-27	Terminal	Didelphic			Japan	Khan et al. (2000)
	5♂♂	2.2-2.6	39-46.5	4.1-4.6	11.5-14	3.2-4.5		46-55	30-32				102-109	10-12		
*silvallus*	4♀♀	1.4-1.9	27-36	4.0-4.3	4.8-5.4	11.3a	62-65	41-42	24-25	27-29	Subventral	Monodelphic			India	Ahmad and Jairajpuri (1983)
*singaporensis*	16♀♀	1-1.23	36-41	3.7-4.2	5.1-6.7	6.3-8.3	59-65	31-33	18-19	29-33	Terminal	Monodelphic			Sigapore	Ahmad et al. (2005)
*southi*	8♀♀	1.8-2.6	31-38	3.7-4.5	6-7	8-11	66-80	46-54	25-31	26-31	Subventral	Monodelphic			India	Dhanam and Jairajpuri (1998)
	1♂	2.2	35	4.3	9	5		52	24	24			76	12		
*stockdilli*	9♀♀	3.3-4.3	41-49	5.2-5.9	7.1-8.8	7.4-10.2	58-73	50-57	32-41	33a	No	Didelphic			New Zealand	Yeates (1988)
	9♂♂	3.2-3.7	45-54	5.1-6	7.9-9.2	6.2-7.5		48-55	28-41				68-70	11-12		
*tarjani*	21♀♀	1.7-2.2	27-41	4.4-5.9	3.3-4.0	16a	47-51	40-43	23-27	23a	No	Didelphic			Nigeria	Mulvey and Jensen (1967)
	2♂♂	1.6-1.7	34-39	4.3-4.4	4			38	23				60-65	?		
	3♀♀	2.1-2.4	34-37	4.1-5.5	4.1-4.5	15-17	51-54	44-47	22-26	19a	No	Didelphic			Cameroon	Siddiqi (2001)
*tenuidentatus*	10♀	1.9-2.5	28-35	4.0-4.5	4.9-5.6	10a	53-57	45-52	27-31	23a	Subventral	Didelphic			Nigeria	Mulvey and Jensen (1967)
*togoensis*	4♀♀	2.2-2.8	41-48	4.8-5.2	4.4-5.9	12.6-17	51.5-59	49-50	23-24	26a	Terminal	Didelphic			Togo	Siddiqi (2001)
*transkeiensis*	2♀♀	1.5-1.6	28-35	4.5	7.3-7.9	6.7a	62	36-38	21-23	31a	No	Didelphic			South Africa	Heyns and Lagerwey (1965)
	6♀♀	1.6-1.7	38-48	3.7-5.7	6.0-7.2	8.7a	56-66	32-35	21-25	36.4a	No	Didelphic			Thailand	Buangsuwon and Jensen (1966)
*trichurus*	?♀	1.2	34.5	4.8	3	15-20	52	28	17						Australia, Fiji, Brazil, Nigeria, Mauritius	Mulvey (1963a), (after Cobb, 1917)
	?♀♀	1.3-2.1	28-46	3.5-5.4	3-5	15-20	53-62	20-35	20-25		Terminal	Monodelphic				Mulvey (1963a)
	?♂	1.7	28	4.4	3.6								78	8-10		
	7♀♀	1.3-1.6	37-41	3.7-4.7	3.4-4	13-18	54-60	26-28	16-18	25-27	Terminal	Monodelphic			India	Jairajpuri (1969)
	1♀	1.7	37	4.6	4	16	61	30	16	26	Subventral	Monodelphic			Costa Rica	Zullini et al. (2002)
*uisongensis*	7♀♀	1.5-1.6	23-26	2.8-3.2	17-19.4	2.0-2.4	67-73	52-63	31-36.5	27-36	No	Didelphic			Korea	Choi and Khan (2000)
	5♂♂	1.5-1.7	24-27	3.2-3.3	19.6-23	1.4-1.6		52-58	30-34				75-92	20-22		
*zullinii*	20♀♀	1.3-2	27-36	3.8-4.7	9.6-13.3	4a	69-76	33-41	22-27	22a	Subdorsal	Monodelphic			Côte	Malcevschi (1981)
	8♂♂	1.3-2	29-37	3.9-4.6	10.3-13	3a		32-37	20-24				68-73	7-10	d’Ivoire	

**Notes:** Measurements in µm excepted *L* in mm. *L* = body length, *a* = body length/maximum body diameter, *b* = body length/pharynx length, *c* = body length/tail length, *c′* = tail length/anal body diameter, *V* = distance from vulva to the anterior end of bodyx100/body length, B.C.L .= buccal cavity length, B.C.W. = buccal cavity width, %DT = dorsal tooth apex length from the base of buccal cavityx100/buccal cavity length, Suppls = ventromedian supllements. ^a^Calculated from original illustrations.

#### Key to species

Currently, 78 species of the genus *Iotonchus* (Cobb, 1917) have been recorded. The following key is based on main characteristics of females.

1. Female genital organ unpaired, prodelphic, or pseudo-prodelphic with rudimentary post genital branch .........................…..….…. 2
  Female genital organ paired, amphidelphic ……….................................................................... 28
2. Genital organ pseudo-prodelphic with rudimantary post genital branch ….........…………. 3
  Genital organ monoprodelphic …………………. 5
3. Small nematode: *L* = 1.4-1.6 mm ...……… *pseudodigonicus* (Ahmad and Jairajpuri, 1983)
  Large nematode: *L*≥2.7 mm …….....………................................................................................……... 4
4. *L* = 2.7-3 mm; smaller buccal cavity = 42-47×27-31 μm .….. *abrahmani* (Loof, 2006)
*L* = 3-3.3 mm, larger buccal cavity = 58-64×32-33 μm …........................…. *kirbyi* (Siddiqi, 1984b)
5. Caudal spinneret absent………...............… 6
  Caudal spinneret present ….……..................… 9
6. Larger body size: *L* = 2.4 mm; larger buccal cavity = 54×30 μm; ♂: unknown ....……......…. *devius* (Siddiqi, 2001)
  Smaller body size: *L*<2 mm …………………... 7
7. *L* = 0.7-1 mm; buccal cavity = 22-24×10-13 μm; post-uterine sac absent; ♂: unknown …................…… *pulsillus* (Loof, 2006)
*L*>1 mm; larger buccal cavity size; post-uterine sac present …….…………......................………. 8
8. Shorter body length: *L* = 1.6 mm; *c* = 7.2-7.9; post-uterine sac 2 body diameter long ……… *lamottei* (Malcevschi, 1981)
  Longer body length: *L* = 1.6-1.9 mm; *c* = 10.3-12.9; post-uterine sac 1 body diameter long ............… *nigeriensis* (Mulvey and Jensen, 1967)
9. Caudal spinneret subterminal ……………… 10
  Caudal spinneret terminal …………………...…19
10. Caudal spinneret subdorsal ………………… 11
  Caudal spinneret subventral …………………... 13
11. Buccal cavity length >50 µm (53-55×30-31) ………….… *sacculatus* (Siddiqi, 2001)
  Buccal cavity length <50 µm ………………… 12
12. Tail 6 anal body diameter long; larger size of buccal cavity = 40-50×28-32 µm ..………..… *acuticaudatus* (Mulvey and Jensen, 1967)
  Tail 4 anal body diameter long; smaller size of buccal cavity = 33-41×22-27 µm …................. …………...…....………...*zullini* (Malcevschi, 1981)
13. Post-uterine sac completely absent …………. 14
  Post-uterine sac present …..…....................... 15
14. Buccal cavity length >40 µm (41-42×24-25 µm); ♂: unknown ……..……..….. *silvallus* (Ahmad and Jairajpuri, 1983)
  Buccal cavity length <40 µm (32-37×28-32 µm); ♂: unknown ……...……. *barqrii* (Jairajpuri, 1969)
15. Post-uterine sac = 3.5 body diameter long; ♂: unknown ……… *recessus* (Yeates, 1992)
  Post-uterine sac <1 body diameter long …… 16
16. *V* = 66-80; buccal cavity = 46-54×25-31 µm … *southi* (Dhanam and Jairajpuri, 1998)
*V*<66 …….............................................................. 17
17. *L* = 2 mm; *V* = 63-64 .….........................................................................… *lacuplanarum* (Yeates, 1992)
*L*<2 mm; *V*<63 ……......................................…... 18
18. *V* = 55-58; buccal cavity = 30-32×15-17 µm ………… *paratrichurus* (Siddiqi, 2001)
*V* = 58-62; buccal cavity = 36-37×19-20 µm …………..................…..… *muneris* (Siddiqi, 2001)
19. *L*>2.5 mm .................................................……… 20
*L*<2.5 mm ………............................................…. 21
20. Buccal cavity length ≥60 µm (buccal cavity = 60-63×37-40 µm); ♂: unknown ............ ...................................... *consimilis* (Cobb, 1917)
  Buccal cavity length ≤60 µm (buccal cavity = 57×40 µm); ♂: unknown ….........................................………. *gymnolaimus* (Cobb, 1893, 1916)
21. Post-uterine sac completely absent ………… 22
  Post-uterine sac present …….......................... 26
22. Shorter body length: *L*≤1.3 mm …………...… 23
  Longer body length: *L*>1.3 mm ….........................................................………. *trichurus* (Cobb, 1917)
23. Buccal cavity length ≤30 µm (22-24×10-13 µm) …………......................................................... *chantaburensis* (Buangsuwon and Jensen, 1966)
  Buccal cavity length >30 µm ………………. 24
24. *V* = 68-72; larger buccal cavity = 36-40×21-29 µm; ♂: unknown ............................………….… …...….................... *nepotum* (Andrássy, 2008)
*V*<68 ….............................................................…. 25
25. Slenderer nematode: *a* = 36-41; smaller buccal cavity = 31-33×18-19 µm; *par refringens vaginae* well developed; tail terminus not swollen; ♂: unknown .….............…..… *singaporensis* (Ahmad et al., 2005)
*a* = 29-31; tail terminus swollen; larger buccal cavity = 34-36×19-20 µm; *par refringens vaginae* absent; ♂: unknown ….......................................... *microdontus* (Thong, 1971)
26. Post-uterine sac<body diameter long; buccal cavity = 40×17 µm; ♂: unknown … ……….. *anisostomus* (Buangsuwon and Jensen, 1966)
  Post-uterine sac>body diameter long; larger buccal cavity size …….………................…….. 27
27. Buccal cavity oval; 50-60×25-31 µm; post-uterine sac = 2.5-3.5 body diameter long ………… *longisaccus* (Mohilal et al., 2000)
  Broader buccal cavity; 58×34 µm; post-uterine sac = body diameter long …............................................................................... *guineae* (Siddiqi, 2001)
28. Caudal spinneret absent …….......……….. 29
  Caudal spinneret present ………..................... 50
29. Tail broadly rounded, hemispheroid, less than 1.5 anal body diameter long ……….... 30
  Tail conoid-arcuate of filiform, longer than 1.5 anal body diameter long ……….….................. 32
30. Larger nematode: *L* = 4.2 mm; buccal cavity = 51×26 µm; tail blunt-conoid, *c′* = 0.7 .........…...…................. *magyar* (Andrássy, 1973)
  Smaller nematodes: *L*≤2.8 mm; tail hemispheroid …...…........................................... 31
31. Slenderer nematodes: *a* = 45-63; smaller buccal cavity = 32-35×23-25 µm; advulval papillae absent ….………... *rotundicaudatus* (Peña-Santiago and Jiménez-Guirado, 1991)
  Stout nematode: *a* = 33; larger buccal cavity = 61×45 µm; advulval papillae present …........................….… *obtusus* (Choi et al., 1999)
32. Tail<6.5 anal body diameter long ……………. 33
  Tail>6.5 anal body diameter long ………….... 42
33. *V*≥67 …...........................................................…. 34
*V*<67 …….............................................................. 37
34. Buccal cavity length >50 µm ……………… 35
  Buccal cavity length <50 µm …………...…… 36
35. Lip region lower (17-20 µm), labial papillae weakly developed; buccal cavity flattened at base …………... *uisongensis* (Choi and Khan, 2000)
  Lip region higher (21-22 µm); labial papillae prominent, protruded; buccal cavity narrowing posteriorly ………....................................................................… *damsanensis* (Choi and Khan, 2000)
36. Large nematode: *L* = 2.2-2.5 mm; slenderer: *a* = 36-43; *c* = 34-41; buccal cavity = 44-48×28-33 µm …………... *aequabilis* (Susulovsky, 1998)
  Smaller nematode: *L* = 1.4-1.8 µm; stout: *a* = 20-23; *c* = 23-28; buccal cavity = 42×30 µm ..……..........… *kirghistanicus* (Sultanalieva, 1983)
37. Vagina without sclerotised pieces ……… *pauli* (Heyns and Lagerwey, 1965)
  Vagina with sclerotised pieces …………....… 38
38. Larger buccal cavity = 57.5-67.5×35-40 µm ...…..…. *paracutus* (Vinciguerra and Orselli, 2000)
  Buccal cavity length <50 µm ……………...… 39
39. Tail>4 anal body diameter long ……………… 40
  Tail<4 anal body diameter long …………........ 41
40. Tail conoid, *c′* = 4.3; advulval papillae present ………………...................…... *acutus* (Cobb, 1917)
  Tail elongated-conoid, *c′* = 5.3-6; advulval papillae absent …........................................................................... .… *hinokumaensis* (Khan et al., 2008)
41. Shorter nematode: *L* = 1.5-1.6 mm; shorter tail: *c* = 24-26; *c′* = 1.7-1.9 ........................……… ….………… *goshiensis* (Khan et al., 2008)
  Larger nematode: *L* = 2-2.3 mm; longer tail: *c* = 18-20; *c′* = 2.7-3.3 …....……...........................................................……… *ogiensis* (Khan et al., 2008)
42. Tail>12 anal body diameter long …………..… 43
  Tail<12 anal body diameter long ……….....… 44
43. Larger nematode: *L* = 2.5-3 mm; vulva posteriorly *V* = 55-60; *c′* = 12; larger buccal cavity = 46-62×36-52 µm ………..…......... *carpathicus* (Popovici, 1990)
  Smaller nematode: *L* = 1.7-2.2 mm; vulva anteriorly: *V* = 47-51; *c′* = 15-17; smaller buccal cavity = 40-43×23-27 µm ……..……….................................................…..… *tarjani* (Mulvey and Jensen, 1967)
44. *L*>3 mm ………………… *stockdilli* (Yeates, 1988)
*L*<3 mm .……...................................................… 45
45. Advulval papillae absent ……………….……… 46
  Advulval papillae present ……………...…...… 48
46. Smaller nematode: *L* = 1.5-1.6 mm; tail≤7 anal body diameter long; vulva posteriorly: *V* = 62; ♂: unknown ……..............................................................… *transkeiensis* (Heyns and Lagerwey, 1965)
  Larger nematode: *L*>1.6 mm; tail>7 anal body diameter long; vulva anteriorly ..........................… 47
47. Slenderer nematode: *a* = 35-41; tail strongly curved, narrowing near terminus; *c′* = 9-10.5; smaller buccal cavity = 38-41×24-28 µm; ♂: unknown .…………….… *arcuatus* (Khan et al., 2000)
*a* = 28-34; tail only slightly ventrally curved; *c′* = 8.8; larger buccal cavity = 40-43×23-29 µm; ♂: unknown ……………..…………...............................………. *clarki* (Mulvey and Jensen, 1967)
48. Vagina without sclerotised pieces ……….… *onchus* (Jairajpuri et al., 2000)
  Vagina with sclerotised pieces .........................… 49
49. Slenderer nematode: *a* = 34-42; vulva posteriorly: *V* = 61-68; smaller buccal cavity = 30-37×25 µm; ♂: unknown ……………..……...… *loteniae* (De Bruin and Heyns, 1992)
*a* = 31; vulva anteriorly: *V* = 59; larger buccal cavity = 54×37 µm; ♂: unknown … ….…….. *globibuca* (Dhanam and Jairajpuri, 2002)
50. Caudal spinneret subterminal …………..... 51
  Caudal spinneret terminal …........................… 58
51. Caudal spinneret subdorsal .................………. 52
  Caudal spinneret subventral ……….……...… 54
52. Tail>14 anal body diameter long …......................................................…..… *mboticus* (Siddiqi, 2001)
  Tail<14 anal body diameter long ……….....… 53
53. *c′* = 7-8; *V* = 57; buccal cavity = 46×22 µm; ♂: unknown …….. *rayongensis* (Buangsuwon and Jensen, 1966)
*c′* = 2.5-4; *V* = 63-69; buccal cavity = 29-40×20-27 µm …............................................................................................... *parageminus* (Jimenez-Guirado, 1994)
54. Advulval papillae absent …………….………… 55
  Advulval papillae present ………………......… 57
55. Longer length of buccal cavity = 57-60 µm ….................… *koupensis* (Siddiqi, 2001)
  Length of buccal cavity<55 µm ………...…… 56
56. Vulva posteriorly: *V* = 57-65…................................……... *indicus* (Jairajpuri, 1969)
  Vulva anteriorly: *V* = 53-57 ...............................…........… *tenuidentatus* (Goodey, 1951; Kreis, 1924)
57. Slenderer nematode: *a* = 50-57; *c′* = 17-18; tail terminus conoid-rounded; caudal pores absent ………...………..… *kilumicus* (Siddiqi, 2001)
*a* = 39-47; *c′* = 8-15; tail terminus broadly rounded; caudal pores present …....................................................................………..… *ndu* (Siddiqi, 2001)
58. Large nematode: *L*>3 mm ….…....................…… 59
  Smaller nematode: *L*<3 mm …….................… 64
59. Length of buccal cavity>65 µm …………… 60
  Length of buccal cavity<65 µm …….......…… 62
60. Advulval papillae absent …..............…… *apapillatus* (Dhanam and Jairajpuri, 1998)
  Advulval papillae present .......................……... 61
61. Larger body length: *L* = 3.8-5.2 mm; slenderer: *a* = 50-59; longer tail: *c* = 4.5-6.3; *c′* = 12-20 ………….....….....…… *lotilabiatus* n. sp.
*L* = 2.7-3.5 mm; *a* = 44-50; shorter tail: *c* = 7.6-8.4; *c′* = 7.5-8.3 ………….........................................................…....… *miamaensis* (Khan and Araki, 2002)
62. Tail shorter: *c* = 17; *c′* = 3-4 .....................…….… *brachylaimus* (Cobb, 1917)
  Tail longer: *c*≤12; *c′*>4 …...………….....................................…...… 63
63. *c* = 10.7-12; *c′* = 4.5-5.2 ……..............................................................…….… *cucumis* (Khan et al., 2002)
*c* = 8-11; *c′* = 6.3 …............................................................................…... *risoceiae* (De Carvalho, 1955)
64. Advulval papillae present …………………...… 65
  Advulval papillae absent ……………..….…… 68
65. *V* = 56-60 .................................................….. *parabasidontus* (Mulvey and Jensen, 1967)
  Vulva posteriorly: *V*>60 .…..…......................… 66
66. Caudal cuticular pores absent …………….……… *nayari* (Mohadas and Prabhoo, 1979)
  Caudal cuticular pores present …...………… 67
67. Length of buccal cavity width = 39-42 µm; Tail shorter: *c* = 10-15; *c′* = 3.6-4.5 ………………...………… *litoralis* (Coetzee, 1967b)
  Length of buccal cavity width = 33-37 µm; *c* = 7-10; *c′* = 5.5-7.8 ….................................................................…… *cuticaudatus* (Jana et al., 2007)
68. *L*<2 mm; small buccal cavity = 29-39×18-24 µm ……..…….. *rinae* (Coetzze, 1967b)
*L*>2 mm; buccal cavity length>39 µm…….... 69
69. Tail short: *c′*  = 3-4 ….....................................................… *geminus* (Heyns and Lagerwey, 1965)
*c′* >4 ……………....................................……..… 70
70. *c′*  = 12.6-17 .................................................................................………….… *togoensis* (Siddiqi, 2001)
*c′* <12 ……..................................................…..… 71
71. *c′*  = 8.3-10; *V* = 51.4-54.4 …................……….…… *aequatorialis* (Vinciguerra and Orselli, 2006)
*c′*<8; *V*>55 …................................................…… 72
72. *V* = 55-60; buccal cavity = 48×30 µm ……… basidontus (Clark, 1961)

*V* = 60-65; buccal cavity = 58-60×38-40 µm …………….............................................…. *sagaensis* (Khan et al., 2000)

### Sequence analysis

Molecular sequences of two individuals of *Iotonchus lotilabiatus* n. sp. were analysed in this study. After sequencing and editing, four sequences were obtained: two 906 to 919 bp length of rRNA (18S), GenBank accession No. MW218936, MW218937 and two 747 bp length D2D3 expansion of rRNA (28S), GenBank accession No. MW218934 and MW228378 were obtained for phylogenetic analyses. This is the first sequences of both 18S and 28S rDNA are provided for a representative of the genus *Iotonchus* and submitted to GenBank. There are only a few sequences of super family Anatonchoidea presented on the Genbank.

A BLAST search for matches to the partial 18S rDNA sequence of new species *Iotonchus lotilabiatus* n. sp. revealed 99% similarity to unidentified species *Miconchus* sp. 1JH–2014 (accession No. KJ636436) and 98% similarity to *Anatonchus tridentatus* (accession No. AJ966474, AY284768). Based on the 28S rDNA sequences, the new species *Iotonchus lotilabiatus* n. sp. only identically 89 to 94% to sequences of *Anatonchus tridentatus* (accession No. AY593065, MG994941).

### rDNA phylogenetic relationships among Mononchida

**Figure 5:**
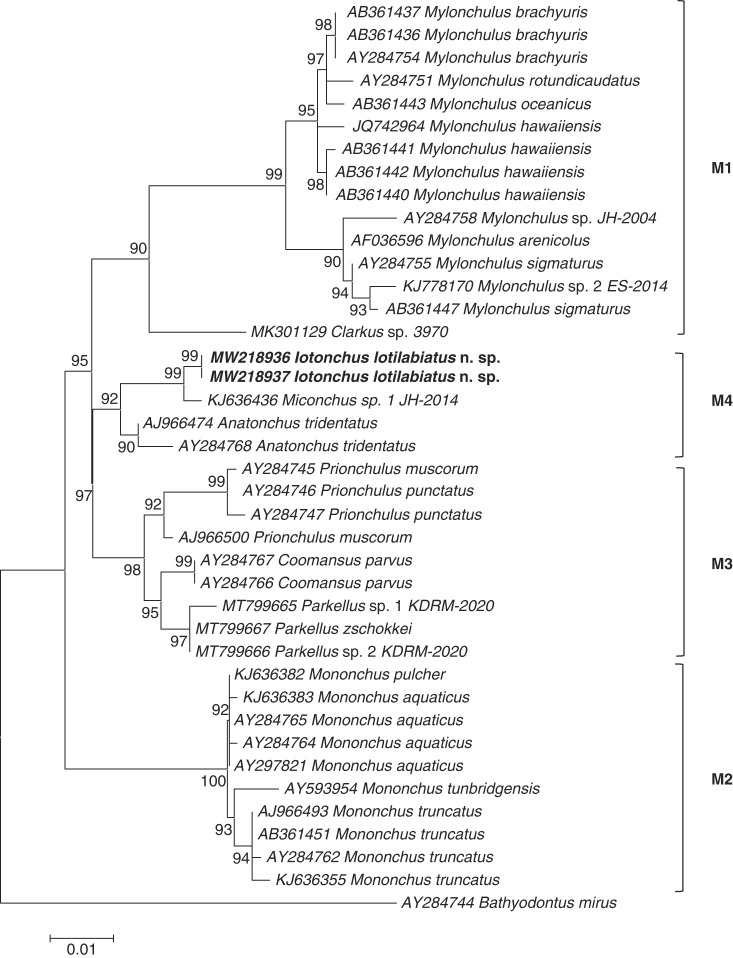
Phylogenetic relationships of *Iotonchus lotilabiatus* n. sp. among other Mononchida, from the analysis of the 18S rDNA sequences under ML (T92+G model (BIC = 5,919.848, AICc = 5,216.912; lnL = ‒2,524.232, G = 0.23, R = 1.78, f(A) = 0.284, f(T) = 0.284, f(C) = 0.216, f(G) = 0.216)). Numbers to the left of the branches are bootstrap values for 1,000 replications.

**Figure 6:**
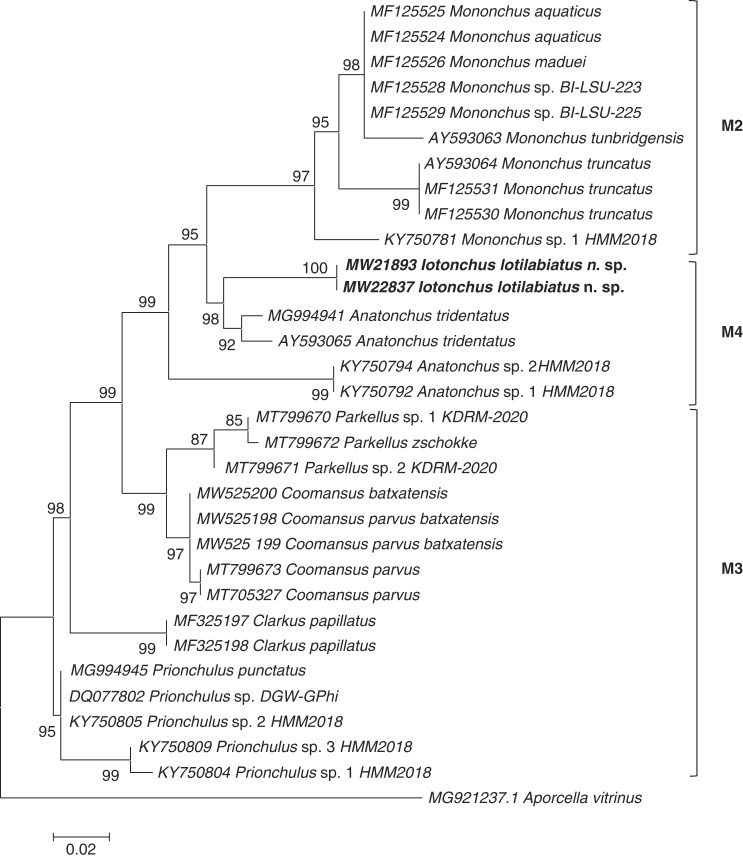
Phylogenetic relationships of *Iotonchus lotilabiatus* n. sp. among other Mononchida, from the analysis of the 28S rDNA sequences under ML (K2+G model (BIC = 2,373.292, AICc = 2,087.600; lnL = ‒1,000.469, G = 0.23, R = 2.00, f(A) = 0.25, f(T) = 0.25, f(C) = 0.25, f(G) = 0.25)). Numbers to the left of the branches are bootstrap values for 1,000 replications.

The results derived from the analyses of these 18S and D2-D3 region of 28S sequences are presented in the molecular trees of Figures 5 and 6, respectively. In both phylogenetic trees, the new species *Iotonchus lotilabiatus* n. sp. were positioned within the M4 clade (following the nomenclature of Holterman et al., 2008, Olia et al., 2008), encompassing representatives of genera *Anatonchus*and *Miconchus* (Anatonchoidea super family) (Figs. 5 and 6). This positioning was confirmed by phylogenetic analyses based on both the 18S and 28S rDNA data.

## Conclusions

During this study, the new species *Iotonchus lotilabiatus* n. sp. is supported by its morphological and molecular characterization. An updated key to species of the genus *Iotonchus* (Cobb, 1916) based on female characteristics and the compendium of all the known species are also supported.
